# Agave proves to be a low recalcitrant lignocellulosic feedstock for biofuels production on semi-arid lands

**DOI:** 10.1186/1754-6834-7-50

**Published:** 2014-04-04

**Authors:** Hongjia Li, Sivakumar Pattathil, Marcus B Foston, Shi-You Ding, Rajeev Kumar, Xiadi Gao, Ashutosh Mittal, John M Yarbrough, Michael E Himmel, Arthur J Ragauskas, Michael G Hahn, Charles E Wyman

**Affiliations:** 1 Department of Chemical and Environmental Engineering, Bourns College of Engineering, University of California, 900 University Ave, Riverside, CA 92507, USA; 2 Center for Environmental Research and Technology, University of California, 1084 Columbia Ave, Riverside, CA 92507, USA; 3 Complex Carbohydrate Research Center, University of Georgia, 315 Riverbend Rd., Athens, GA 30602, USA; 4 Institute of Paper Science and Technology, Georgia Institute of Technology, 500 10th St. NW, Atlanta, GA 30332, USA; 5 National Renewable National Laboratory, 15013 Denver W Pkwy, Golden, CO 37831, USA; 6 BioEnergy Science Center, Oak Ridge National Laboratory, 1 Bethel Valley Rd, Oak Ridge, TN 37831, USA; 7 Current address: DuPont Industrial Biosciences, 925 Page Mill Rd, Palo Alto, CA 94304, USA; 8 Current address: Energy, Environmental & Chemical Engineering Department, Washington University in St. Louis, 1 Brookings Drive, St. Louis, MO 63130, USA

**Keywords:** Agave, Biofuels, Feedstock, Low recalcitrance, Semi-arid land

## Abstract

**Background:**

Agave, which is well known for tequila and other liquor production in Mexico, has recently gained attention because of its attractive potential to launch sustainable bioenergy feedstock solutions for semi-arid and arid lands. It was previously found that agave cell walls contain low lignin and relatively diverse non-cellulosic polysaccharides, suggesting unique recalcitrant features when compared to conventional C_4_ and C_3_ plants.

**Results:**

Here, we report sugar release data from fungal enzymatic hydrolysis of non-pretreated and hydrothermally pretreated biomass that shows agave to be much less recalcitrant to deconstruction than poplar or switchgrass. In fact, non-pretreated agave has a sugar release five to eight times greater than that of poplar wood and switchgrass . Meanwhile, state of the art techniques including glycome profiling, nuclear magnetic resonance (NMR), Simon’s Stain, confocal laser scanning microscopy and so forth, were applied to measure interactions of non-cellulosic wall components, cell wall hydrophilicity, and enzyme accessibility to identify key structural features that make agave cell walls less resistant to biological deconstruction when compared to poplar and switchgrass.

**Conclusions:**

This study systematically evaluated the recalcitrant features of agave plants towards biofuels applications. The results show that not only does agave present great promise for feeding biorefineries on semi-arid and arid lands, but also show the value of studying agave’s low recalcitrance for developments in improving cellulosic energy crops.

## Background

A large cellulosic biomass supply will be critical to establishing a lignocellulosic industry with a major long term impact on sustainably supplying fuels and chemicals [1,2]. However, the high water demands of many plants would limit fuel production to regions with high annual rainfall or irrigation that would eventually compete with growing food [3]. Thus, conversion of drought-resistant cellulosic feedstocks (such as agave) to biofuels would expand energy crop production to semi-arid lands that occupy about 18% of the terrestrial surface [1,3,4]. By using the Crassulacean Acid Metabolism (CAM) pathway, agave has high biomass productivity with minimal inputs of water and nutrients [5]. In addition, agave offers environmental attributes such as preventing desertification and removing heavy metals ions from contaminated soil [6]. These attractive features make agave potentially valuable as a low-cost global biofuels feedstock [7].

Our recent study showed agave cell walls contain relatively low amounts of lignin and a diverse range of non-cellulosic polysaccharides (Additional file 1 shows this in more detail) when compared to most woody and herbaceous plants [8]. As lignin and non-cellulosic cell-wall structural polysaccharides shield cellulose microfibrils from enzymes [9,10], lower amounts of these in agave suggest a low cell wall recalcitrance, in other words, agave has a potential of high sugar release following pretreatment and/or enzymatic hydrolysis. However, although overcoming biomass recalcitrance is the primary roadblock to low cost biofuels [1,11], little is known about the susceptibility of structural carbohydrates in agave species to sugar release. Thus, based on the sugar composition in agave fiber that we determined in earlier research [8], this paper presents a detailed study on the enzymatic saccharification of agave bagasse samples with or without hydrothermal pretreatment. In addition, important agave cell wall structural characteristics other than fermentable sugar composition, such as interactions of non-cellulosic wall components, cell wall hydrophilicity, and enzyme accessibility are also studied and reported here to better understand the effects of agave cell wall structure on its sugar release performance following pretreatment and/or enzymatic hydrolysis. By comparing agave to other lignocellulosic feedstock (poplar and switchgrass), the results of this paper provided valuable insights in determining the feasibility of agave as an energy crop for arid and semi-arid lands. Furthermore, understanding the unique cell wall features of agave that influence its low recalcitrance against enzymatic cell wall deconstruction may provide valuable insights for improving sugar release in other plants.

In this study, we prepared four biomass samples from the leaves and/or hearts of three popular agave species: *Agave americana* leaves (AAL), *Agave salmiana* leaves (ASL), *Agave tequilana* leaves (ATL), and *Agave americana* heart (AAH), as leaves and heart are the main portions of agave to be utilized as cellulosic feedstocks . *A. americana* and *A. salmiana* were chosen because they are common in most countries and have high productivity [4,7]. *A. tequilana* was selected because it is widely cultivated in Mexico for tequila, with most of the leaves and heart bagasse left as waste that could be used as feedstocks for biofuels production [12]. Two leading energy crop candidates, poplar (*Populus trichocarpa*) and switchgrass (*Panicum virgatum*), were subjected to the same procedures to provide a perspective on agave recalcitrance.

## Results and discussion

### Enzyme formulations

We first determined how different enzyme activities can affect the biological deconstruction of biomass in order to identify enzyme formulations that increased sugar release from the several agave species, poplar, and switchgrass. Seven fungal enzyme cocktails, which contain different proportions of cellulase, xylanase, hemicellulase and pectinase activities, were prepared from commercial Genencor (now DuPont Industrial Biosciences Palo Alto, CA, USA) biomass enzymes at the same total protein loadings, including Accellerase 1500, Accellerase XY, Accellerase XC, and Multifect Pectinase (details of these seven fungal enzyme cocktails are listed in Additional file 2). The results showed that supplementing Accellerase 1500 with Accellerase XC and especially Multifect Pectinase increased sugar release from AAL, ASL and AAH (Additional file 3a, b and d), while Accellerase XY supplementation increased sugar release from ATL (Additional file 3c). Although multiple active enzymes such as xylanase or hemicellulase are important to achieve a high sugar yield for pretreated poplar and switchgrass [13], supplementations of Accellerase XY, Accellerase XC, or Multifect Pectinase to Accellerase 1500 did not have as significant an impact for these two species as it did for agave (Additional file 3e and f). Overall, the enzyme cocktail designated “1500 + XY + *P*” (Additional file 2) provided the highest total sugar release for all samples (Additional file 3a-f) and was applied for all subsequent experiments.

### Composition and extractability of non-cellulosic wall components

**Figure 1:**
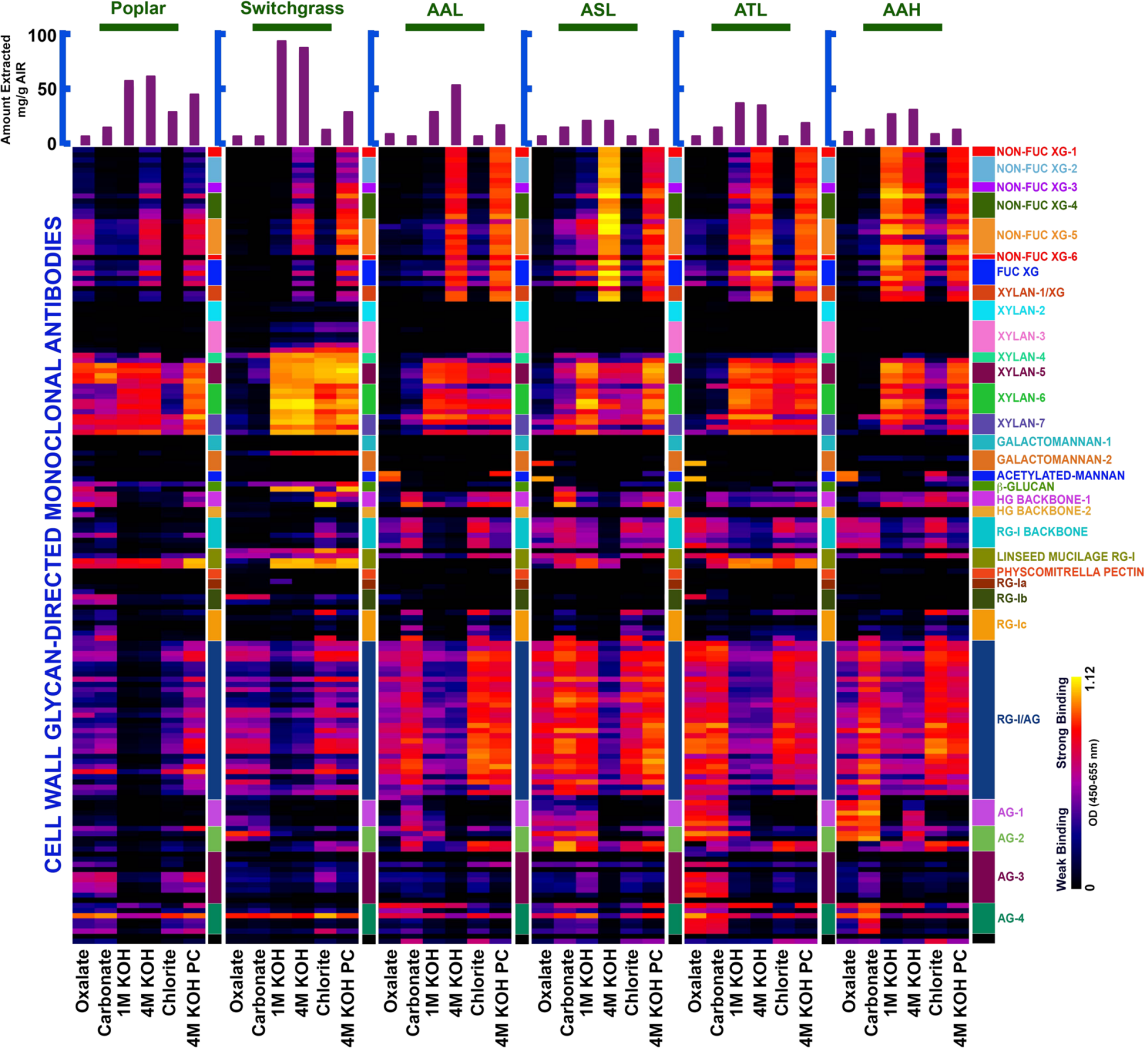
**Glycome profiling of untreated *P. trichocarpa *(Poplar), *P. virgatum *(Switchgrass), *A. americana *leaves (AAL), *A. salmiana *leaves (ASL), *A. tequilana *leaves (ATL), and *A. americana *heart (AAH) biomasses.** Sequentially extracted materials released from each biomass sample by various reagents (as labeled at the bottom of each map) were loaded onto the ELISA plates and screened against an array of plant glycan-directed monoclonal antibodies. The legend panel on the right displays the nature of the polysaccharides predominantly recognized by these monoclonal antibodies (mAbs). Antibody binding is represented as colored heat maps, with black signifying no binding, and light yellow representing the strongest binding. The bar graphs at the top indicate the amount of material recovered at each extraction step per gram of alcohol insoluble residue (AIR). AAL: *A. americana* leaves; ASL: *A. salmiana* leaves; ATL: *A. tequilana* leaves; AAH: *A. americana* heart; AG: Arabinogalactan; HG: Homogalacturonan; KOH: Potassium Hydroxide; PC: Post Chlorite; RG: Rhamnogalacturonan; XG: Xyloglucan.

The variation in enzyme formulations that are most effective in deconstructing different biomass materials suggests cell walls of agave species are significantly different from those of poplar and switchgrass in terms of complex non-cellulosic polysaccharide types. Thus, glycome profiling [14] was used to better understand cell wall properties, as well as important cell wall components of agave species that contribute to biomass recalcitrance, with the results compared to those of poplar and switchgrass. Glycome profiling [14] uses a comprehensive suite of plant glycan-directed monoclonal antibodies (mAbs) to monitor the composition, structure, and extractability of most major non-cellulosic polysaccharides. Antibodies that recognize epitopes on xyloglucan, pectin (including mAbs in the RG-I backbone, RG-I/AG, AG-1 and AG-2 groups), and xylan showed strong binding to fractions extracted from agave cell walls, while xylan epitopes predominated in the glycome profiles of poplar and switchgrass (Figure 1). These results demonstrate the presence of multiple non-cellulosic polysaccharides, especially pectin, in agave leaves and hearts. The presence of these non-cellulosic polysaccharides in agave is consistent with improved sugar yields from agave that result from the inclusion of enzymes such as hemicellulase and pectinase in the digestion cocktails. In addition, wall components extracted by less harsh chemical reagents (oxalate, carbonate) accounted for a relatively greater proportion of total extractives from agave materials, while the amounts of wall components associated directly with lignin (chlorite extract) and secured within the walls by lignin (4 M KOH PC extract) were significantly lower in agave than in poplar and switchgrass (Figure 1). Together with the low lignin content in agave (Additional file 1), the high extractability of non-cellulosic cell wall components indicates relatively low levels of resistance (outside of cellulose microfibrils) against the enzymatic degradation of agave cell walls, which in turn suggests that agave is less recalcitrant than poplar and switchgrass.

### Enzymatic digestibility of non-pretreated and hydrothermal pretreated biomasses Reject, please keep it as biomass, not biomasses

**Figure 2:**
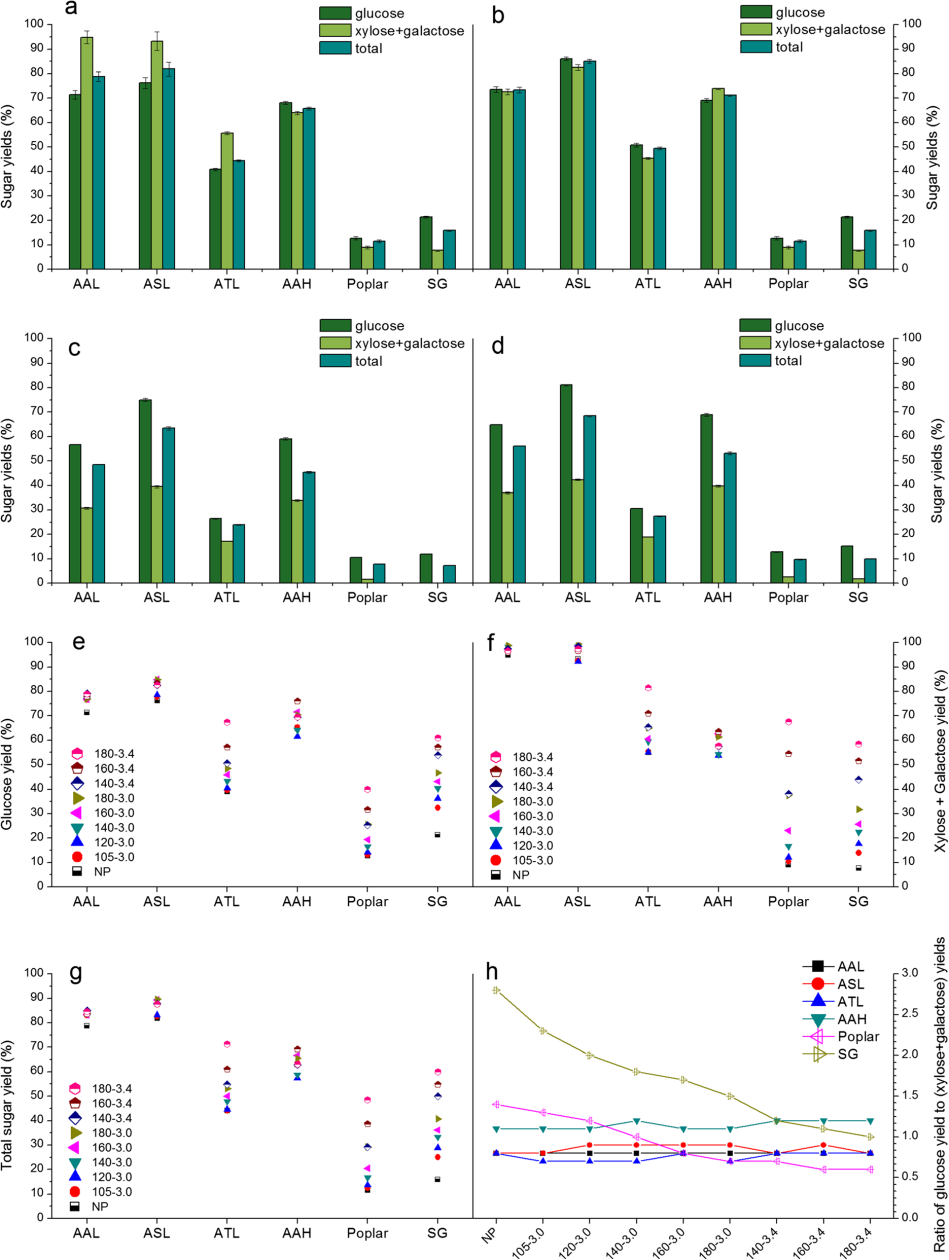
**Sugar yield data from enzymatic hydrolysis of (a,c,d) non-pretreated (b) extractives free non-pretreated and (e-h) hydrothermal-pretreated *P. trichocarpa *(Poplar), *P. virgatum *(Switchgrass: SG), *A. americana *leaves (AAL), *A. salmiana *leaves (ASL), *A. tequilana *leaves (ATL), and *A. americana *heart (AAH) biomasses.** Biomass samples were digested with cellulase supplemented with xylanase and pectinase as described in the Materials and Method Section: **(a,b,e-h)** 72 hours hydrolysis using 150 mg protein/g structural sugar enzyme loading, **(c)** 72 hours hydrolysis using 15 mg protein/g structural sugar enzyme loading, **d)** 144 hours hydrolysis using 15 mg protein/g structural sugar enzyme loading. Hydrothermal pretreatment conditions are described in Supporting Information S4. Pretreatment conditions 105 to 3.0, for example, represents pretreatment at 105°C with a severity factor of 3.0; and NP represents no pretreatment. Yields reflect the amount of sugar released of the maximum available in raw biomass. Error bars represent standard deviation of quadruplicates. AAL: *A. americana* leaves; ASL: *A. salmiana* leaves; ATL: *A. tequilana* leaves; AAH: *A. americana* heart; NP: Non-pretreated; SG: Switchgrass.

Based on these findings, we enzymatically hydrolyzed non-pretreated biomass using the optimized enzyme formulation, in order to quantitatively determine biomass recalcitrance. We found that non-pretreated agave biomass achieved dramatically higher sugar yields than non-pretreated poplar or switchgrass at both low, and high enzyme loadings of 15 mg and 150 mg total protein/g structural carbohydrate in raw biomass, respectively (Figure 2a, c and d). In fact, the best ASL samples were able to release about 80% of total cell wall carbohydrates at the high enzyme loading of 150 mg total protein/g structural carbohydrate in raw biomass. Although the xylose + galactose yield dropped at the low enzyme loading of 15 mg total protein/g structural carbohydrate in raw biomass, glucose yield up to 81.1% was still able to be realized for ASL. To confirm this significant finding, we applied the same enzymatic hydrolysis conditions to agave samples that had been sequentially extracted with water and ethanol to avoid potential interference or skewing of yield data from free sugars in raw biomass. This allowed us to focus on those sugars that are released by deconstruction of structural polysaccharides. The resulting extractives free materials showed consistently higher sugar releases from agave than from poplar (about 4.3 to 7.3 times higher, Figure 2b) or switchgrass (about 5.3 to 3.1 times higher, Figure 2b). These results confirmed that agave species have significantly lower recalcitrance to biological deconstruction than other lignocellulosic biomass being studied as biofuels feedstocks.

Utilizing a much less recalcitrant lignocellulosic feedstock would dramatically reduce the production costs of advanced biofuels through using mild pretreatment conditions and low enzymes doses [2,15,16]. Thus, a series of low-severity hydrothermal pretreatments (detailed pretreated conditions are listed in Additional file 4) were applied to further understand differences in plant recalcitrance that affect sugar release in pretreatment and enzymatic hydrolysis. At the same pretreatment severity [17], higher temperatures resulted in higher sugar yields than pretreatments with longer reaction times (Figure 2e, f and g), indicating that pretreatment temperature has a greater impact on biomass digestibility than does reaction time. Increasing the severity of the pretreatment significantly increased the enzymatic digestibility of pretreated poplar and switchgrass (Figure 2e, f and g). However, the impact of tested pretreatment conditions on sugar yields from agave species was very limited, especially AAL, ASL, and AAH samples. These results suggest that pretreatment for agave is not as critical as for conventional lignocellulosic feedstocks to overcome agave recalcitrance. Thus, the economic tradeoffs between a slight sugar yield increase must be weighed against additional pretreatment costs. Another interesting difference between agave, poplar and switchgrass is the ratio of glucose yield to ‘xylose + galactose’ yield over the pretreatment conditions. The decreasing trends in such ratios for poplar and switchgrass indicate that increasing pretreatment severity improves digestibility of hemicellulose more than cellulose (Figure 2h), as expected in that hemicellulose is relatively loose and protects crystalline cellulose. In contrast, however, the corresponding ratios for agave materials stayed nearly constant (Figure 2h). This interesting difference suggests that agave cellulose, as well as more easily hydrolyzed hemicellulose and pectin, was disrupted to a similar extent over the full range of pretreatment conditions. Thus, agave cell walls must have unique features when compared to other biomasses that increase cellulose digestibility.

### Structural characterization of cell walls

**Figure 3:**
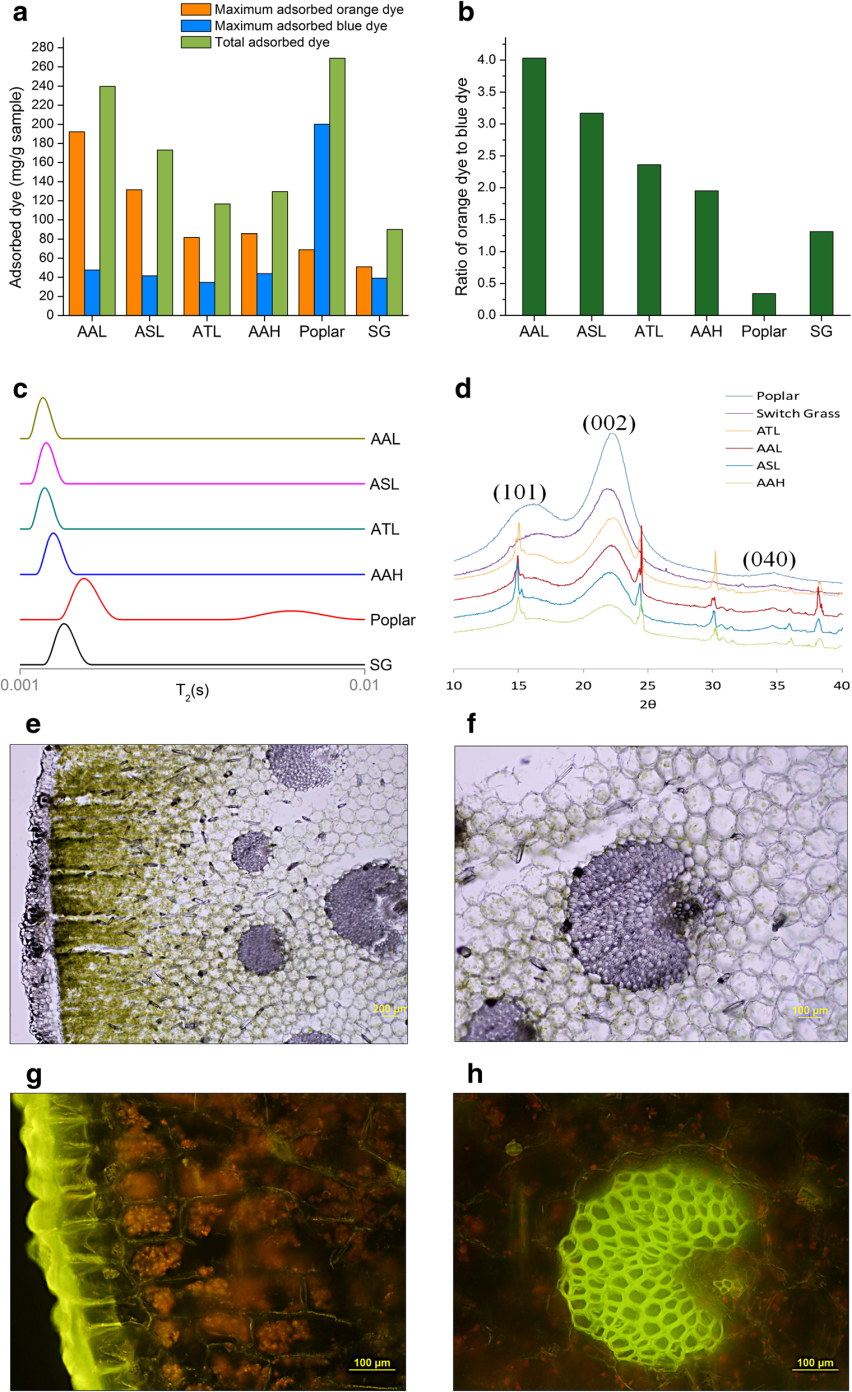
**Structural characterization of non-pretreated *P. trichocarpa *(Poplar), *P. virgatum *(Switchgrass: SG), *A. americana *leaves (AAL), *A. salmiana *leaves (ASL), *A. tequilana *leaves (ATL), and *A. americana *heart (AAH) biomasses. (a)** Simons’ Stain results for biomass pore surface area represented by the amount of absorbed dye, mg dye/g of sample. **(b)** Simons’ Stain results for relative enzyme accessibility represented by ratio of absorbed large dye to small dye, [mg orange dye/g sample]/[mg blue dye/g sample]. **(c)** Spin-spin relaxation times (T_2_) of absorbed water within biomass samples produced via ILTs of CMPG T_2_ experiments. **(d)** XRD spectrum. Confocal laser scanning microscopy of AAL cell walls: bright field **(e,f)** and auto-fluorescence **(g,h)**. AAL: *A. americana* leaves; ASL: *A. salmiana* leaves; ATL: *A. tequilana* leaves; AAH: *A. americana* heart; SG: Switchgrass.

To gain better insight into agave structural characteristics that may enhance cell wall reactivity, we applied the Simons’ Stain test to provide insights into the pore surface area (Figure 3a) and relative accessibility (Figure 3b) of biomass samples. The results showed that agave had a more accessible surface area (amount of adsorbed large dye: orange dye) and higher relative accessibility (ratio of adsorbed large to small dye: orange to blue dye) than poplar and switchgrass, especially for samples of AAL and ASL, in strong agreement with the sugar release results presented above. As high enzyme accessibility of raw agave materials enables enzymes to more easily hydrolyze cell wall polysaccharides without pretreatment, this finding helps explain why the sugar yield from agave was not as sensitive to pretreatment conditions as poplar and switchgrass. Next, water mobility in biomass cell walls was monitored by measuring the ^1^H NMR distribution of spin-spin relaxation times (T_2_) of absorbed water. Agave samples showed shorter T_2_ values than switchgrass or poplar (Figure 3c), indicating stronger cell wall interactions with water molecules, in other words, higher hydrophilicity that facilitates mass transfer and cell wall reactivity in water media during pretreatment and enzymatic hydrolysis. Furthermore, X-Ray Diffraction (XRD) was applied to qualitatively compare the ordered structure of cellulosic materials in this study. The XRD spectrum of the Avicel PH 101 cellulose showed diffraction peaks of cellulose I, corresponding to (101), (002), and (040) lattice planes (see details in Additional file 5). Comparing typical peaks of agave samples to those of poplar and switchgrass showed that agave cell walls had less well-defined crystalline structure as cellulose I than most lignocellulosic biomass (Figure 3d). In fact, agave heart bagasse, AAH, was even more amorphous than six-hour ball milled Avicel (Additional file 5).

These unique structural characteristics of agave species discussed above provide valuable insights in explaining why its sugar release patterns from pretreatment and enzymatic hydrolysis are so different from poplar and switchgrass, and why the recalcitrance of agave is unusually low. In addition, we find it useful to postulate how these low recalcitrant features of agave cell walls could relate to how agave plants survive in arid regions. For example, the thick, green agave leaves serve in both photosynthesis and water storage and accommodate large, thin-walled parenchyma and collenchyma cells as succulent water-storing tissues [18]. Confocal laser-scanning microscopy confirmed that parenchyma cells that possess non-lignified primary walls contributed the majority of agave mass (Figure 3e, f, g and h), in contrast to lignified sclerenchyma cells (secondary cell walls) that dominant conventional lignocellulosic feedstocks. Another example is that during its reproductive growing stage, the agave plant is believed to extract polysaccharides from its vegetative and storage organs to produce its flower stalk, leaving the leaves yellow, thin, and dry. This physiological phenomenon suggests that the defensive cell wall structure in woody and grass biomass against biological deconstruction might not be beneficial to agave plants that need to hydrolyze polysaccharides in order to provide energy for reproduction. Instead, a thick layer of cuticle structure was found on the outer layer of the epidermis cells (Figure 3e and g), which may help to prevent water loss and to protect against microbial attack in the natural environment. The possible associations of such special plant characteristics with cell wall structural features provide directions to discover, identify, and develop new, advanced low recalcitrant energy crops.

## Conclusions

In summary, we discovered and demonstrated that agave is a low recalcitrant material that could expand production of biofuels to arid and semi-arid lands, and dramatically reduce processing costs. Furthermore, we have shown that its low recalcitrance arises from several key features such as: a loose non-cellulosic wall component structure, high enzyme accessibility, good hydrophilicity, and less ordered crystalline structure. Further understanding as to how agave species control such traits could provide valuable insights to greatly facilitate the development of low recalcitrant, highly productive, and drought resistant biomasses. Thus, future biorefineries might benefit from a much less recalcitrant lignocellulosic biomass that can be grown with much less water on semi-arid and arid lands not suitable for producing food.

## Materials and methods

### Plant materials

AAL, ASL, ATL, and AAH were collected fresh from the San Jose area (California, USA) and prepared at UCR University of California, Riverside, as described in detail elsewhere [8]. Poplar and switchgrass were grown at Oak Ridge National Laboratory (ORNL) and provided through the BioEnergy Science Center (BESC). Dry biomass samples were knife milled through a 40-mesh (425 μm) screen prior to experiments.

### Compositional analysis

The composition of agave samples was determined according to National Renewable Energy Laboratory (NREL) standard biomass analysis procedures and reported elsewhere [8]. For poplar and switchgrass, the glucan and xylan contents were determined using unwashed biomass.

### Pretreatment and enzymatic hydrolysis

Pretreatment and/or enzymatic hydrolysis was performed in a high throughput pretreatment and enzymatic hydrolysis (HTPH) system [19-21], using a customized 96-well plate reactor. Dry biomass weighing 4.5 mg was added to each well using an automated solid and liquid dispensing robotics platform (Core Module II, Freeslate Inc., Sunnyvale, CA, United States) followed by 445 μL of deionized (DI) water. The well plates were then clamped together and placed in a custom-built steam chamber for pretreatment, as described in detail elsewhere [19]. Following pretreatment, 30.5 μL of a mixture of citric acid buffer (1 M, pH 4.8), sodium azide (10 g/L), and dilute enzyme mixture was added to each well, and the plates were incubated at 50°C in a Multitron shaker (Multitron Infors-HT, ATR Biotech, MD) at 150 rpm for 72 hours. The well-plates were then centrifuged at 2700 rpm for 30 minutes and the liquid hydrolyzate was transferred to HPLC vials for analysis. All enzymatic hydrolysis experiments were performed in quadruplicate. Sugar concentrations were determined by a Waters Alliance e2695 HPLC with a 2414 refractive index (RI) detector (Waters Corporation, Milford, MA, United States) and a BioRad Aminex HPX-87H column (Bio-Rad Life Science, Hercules, CA, United States).

### Enzyme loading and formulation

A high protein loading of 150 mg/g structural carbohydrates in raw materials was applied, using Genencor enzymes (DuPont Genencor Science, Palo Alto, CA, United States): cellulase (Accellerase 1500, Lot No.:1681198062), xylanase (Accellerase XY, Lot No.:4901131618), xylanase (Accellerase XC, Lot No.:4861066335), and pectinase (Multifect Pectinase, Lot No.:4861295753). Enzyme formulations are listed in Additional file 2.

### Enzymatic hydrolysis of non-pretreated biomass

The citric acid buffer, sodium azide, and diluted enzyme mixture was added to each well without taking the plates through pretreatment. A protein loading of 15 and 150 mg/g structural carbohydrates in raw materials was used with mass ratio of Accellerase 1500, Accellerase XY, Multifect Pectinase is 6:1:1. The 15 mg/g structural carbohydrates enzyme loading experiments were run for both 72 and 144 hours.

### Low-severity hydrothermal pretreatment and enzymatic hydrolysis

A series of relatively mild hydrothermal pretreatments were conducted at the conditions listed in Additional file 4. After pretreatment, the 150 mg/g enzyme protein loading and formulation were applied as above.

### Glycome profiling

Glycome profiling is an ELISA-based method that uses plant glycan-directed monoclonal antibodies (mAbs) to identify cell wall carbohydrate components present in sequential cell wall extracts prepared with increasingly harsh chemical reagents [14,22,23]. About 250 mg (dry weight) of each non-pretreated agave, poplar, and switchgrass samples were sequentially washed with absolute ethanol and acetone to remove extractives. The washed residues were then vacuum-dried overnight and subjected to extraction steps in 10 mg mL^-1^ suspensions based on the starting dry biomass weight used. Firstly, the biomass was suspended in 50 mM ammonium oxalate (pH = 5.0) and incubated overnight with constant mixing at room temperature. After incubation, the mixture was centrifuged at 3400 g for 15 minutes, and the resulting supernatant was decanted and saved as the oxalate fraction. Following the same protocol, the pellet was then subjected to additional sequential extractions using, in turn, 50 mM sodium carbonate (pH 10) containing 0.5% (w/v) weigh by volume sodium borohydride, and 1 M KOH, 4 M KOH, each containing 1% (w/v) sodium borohydride. The pellet remaining after the 4 M KOH extraction was then treated with sodium chlorite (100 mM) to breakdown lignin polymers into smaller components, as described previously [14]. Lastly, the pellet left following sodium chlorite treatment was subjected to a final extraction with 4 M KOH containing 1% (w/v) sodium borohydride to extract material that had previously been secured within the walls by lignin (4 M KOH PC). The resulting residual pellet was not analyzed any further. The 1 M KOH, 4 M KOH, and 4 M KOH PC extracts were neutralized with glacial acetic acid. All extracts were dialyzed against four changes of DI water (with an approximate sample to water ratio of 1:60) for 48 hours at room temperature and subsequently lyophilized. After estimating the total sugar contents of the cell wall extracts using the phenol-sulfuric acid method, the extracts were dissolved in DI water to a concentration of 0.2 mg mL^-1^. Next, all extracts were diluted to the same sugar concentration of 20 μg mL^-1^ for loading onto ELISA plates. Diluted extract (50 μL) was added to each well and allowed to evaporate overnight at 37°C until dry. The ELISAs were conducted as described using an array of 155 monoclonal antibodies specific to epitopes from most major groups of plant cell wall polysaccharides [14]. Negative controls consisting of water blanks without antigen were included in all assays and their absorbance subtracted from all samples. None of the monoclonal antibodies that were used show background in the ELISA assays. ELISA data are presented as heat maps in which antibodies are grouped based on a hierarchical clustering analysis of their binding specificities against a diverse set of plant glycans [14]. Monoclonal antibodies used in this study (see details in Additional file 6) were obtained from the Complex Carbohydrate Research Center collection (available through CarboSource Services).

### Simons’ stain

A modified Simons’ stain assay based on previously developed procedures was applied [24]. D_O_ (Pontamine Fast Orange 6RN) and D_B_ (Pontamine Fast Sky Blue 6BX) dyes were obtained from Pylam Products (Garden City, NY, United States). First, 1% (w/v) orange dye solution was poured into an Amicon EMD Millipore Corporation, Billerica, MA, USA ultrafiltration apparatus and filtered through a 100 K ultrafiltration membrane under 28 psi nitrogen gas pressure [25], until 20% of the original solution was left. 1.0 mL of the retained dye solution in the filter was dried in a 50°C vacuum oven for five days, and the weight of the solid residue was then measured to determine the concentration of the filtered solution. The result was then used to calculate dilution with the filtered orange dye solution to the required concentration (10 mg mL^-1^) for Simons’ staining. Next, 100 mg of biomass samples were weighed into five 15 mL centrifuge tubes, followed by adding 1.0 mL of phosphate buffered saline solution (pH 6, 0.3 M PO_4_, 1.4 mM NaCl). Then, both D_O_ solution (10 mg mL^-1^) and D_B_ solution (10 mg mL^-1^) were added in increasing volumes (0.25, 0.50, 0.75, 1.0, 1.5 mL) to the five tubes containing biomass sample and buffer to create a 1:1 mixture of D_O_ and D_B_ dyes at increasing concentrations. Following that, DI water was added to each tube to make the final volume 10.0 mL. The tubes were incubated at 70°C with shaking at 200 rpm for six hours and then centrifuged at 10,000 rpm for eight minutes. After that, ultraviolet (UV) absorbance of supernatant was measured on a Lambda 35 UV–vis spectrophotometer (PerkinElmer, Waltham, MA, United States) at 455 nm and 624 nm. The concentration of the D_O_ and D_B_ dyes (C_O_ and C_B_, respectively) in the supernatant was calculated using the following two equations (based on Lambert-Beer law for a binary mixture) [25]:

(1) $$A_{455\mathrm{nm}}=\varepsilon_{O}/455LC_{O}+\varepsilon_{B}/455LC_{B}$$

(2) $$A_{624\mathrm{nm}}=\varepsilon_{O}/624LC_{O}+\varepsilon_{B}/624LC_{B}$$

The extinction coefficients ε_O_ and ε_B_ were determined by preparing standard calibration curves at 455 and 624 nm. The amount of dye adsorbed by the biomass was then calculated by subtracting the amount of dye in the supernatant from the added amount initially. Total adsorption is reported as mg of dye per gram of biomass.

### Water mobility

Biomass samples were conditioned in a sealed desiccator at 25°C and approximately 100% relative humidity over a 0.01 (w/v) NaN_3_ solution for seven days. The moisture contents in all samples were found to be 26 ± 3%. ^1^H spin-spin (T_2_) NMR measurements were carried out on a Bruker DSX-300 spectrometer (Bruker BioSpin Corporation, Billerica, MA, USA), operating at frequencies of 300.13 MHz for ^1^H in a Bruker static probe. The spin-spin relaxation times were determined using a standard two dimensional Carr-Purcell-Meiboom-Gill (CPMG) sequence with a 5 μs (90°) ^1^H pulse, 10 μs (180°) ^1^H pulses, 16 scans, 10 s recycle delay and τ = 0.0002 s. 16 data points were recorded between n = 4–1024 echoes (0.00164 – 0.41984 s). Inverse Laplace transforms (ILT) were determined by the Matlab 7.13 program written by P. T. Callaghan at Victoria University of Wellington (Wellington, New Zealand) to process one and two dimensional ASCII data measuring either diffusion or relaxation characteristics of heterogeneous proton systems. This program is based on unconstrained regularization, non-negative least squared fit, and singular value decomposition algorithms. The routine was tested using a series of multi-exponential and stretched-exponential functions of varying component weights, widths, and characteristic decay times demonstrating fairly good accuracy, resolution and stability in the corresponding distributions produced. To assess the effect of noise, relaxation curves were generated using a multi-exponential function, and each data point was allow to increase or decrease by a maximum of 10%. The variance at each data point was controlled by a random number generator to simulate a randomly noisy relaxation curve. The resulting transforms produced reliable peak intensities, positions, and widths. A common technique to extract information for comparison on systems having wide distributions of nuclear relaxers or T_2_ decays utilizes an ILT routine [26,27].

### X-ray diffraction (XRD)

X-ray diffraction (XRD) was performed to evaluate the crystalline structure of biomass samples by using a Rigaku (Tokyo, Japan) Ultima IV diffractometer with CuKα radiation having a wavelength of λ (Kα1) = 0.15406 nm generated at 40 kV and 44 mA. The diffraction intensities of air dried samples placed on a quartz substrate were measured in the 2θ range of 8 to 42° using a step size of 0.02° at a rate of 2°/min.

### Confocal laser scanning microscopy

A confocal laser scanning microscope (Nikon ECLIPSE E800 microscope equipped with the Nikon C1 confocal system) (Nikon Instruments Inc. Melville, NY, USA) was used for imaging the fresh cut transverse section of agave leaf. Images of white light and auto-fluorescence were excited by a 488 nm laser and detected by 515/30 nm emission filter. All images were recorded at a resolution of 4048 × 3027 pixels.

## Abbreviations

AAL: *Agave americana* leaves; ASL: *A salmiana* leaves; ATL: *A tequilana* leaves; AAH: *A americana* heart; BESC: BioEnergy Science Center; CAM: Crassulacean Acid Metabolism; DI: Deionized; HTPH: High throughput pretreatment and enzymatic hydrolysis; ILT: Inverse laplace transforms; NMR: Nuclear magnetic resonance; NREL: National Renewable Energy Laboratory; ORNL: Oak Ridge National Laboratory; RI: Refractive index; XRD: X-Ray Diffraction.

## Competing interests

Michael Himmel and Charles Wyman are Editors-in-Chief of Biotechnology for Biofuels. All authors declare that they have no other competing interests.

## Authors’ contributions

HL, RK, SP, MBF, SYD, MEH, AJR, MGH and CEW designed the research approach; HL, SP, MBF, SYD, XG, AM, and JMY performed the research; all authors analyzed or interpreted data; all authors drafted or provided important edits to various parts of the paper; HL, RK, MGH and CEW revised the overall paper. All authors read and approved the final manuscript.

## Supplementary Material

Additional file 1 **Chemical composition of agave, poplar, and switchgrass.** A table lists chemical composition data of different agave samples, as well as poplar and switchgrass. Click here for file

Additional file 2 **Enzymes, formulations, and protein proportions of fungal enzyme cocktails applied for biomass hydrolysis.** A table lists composition of enzyme cocktails which were used in the experiment for enzymatic hydrolysis of biomass samples. Click here for file

Additional file 3 **Total sugar release from hydrothermal pretreatment (180C- 11.1 min) followed by enzymatic hydrolysis of (a) *A. americana* leaves (AAL), (b) *A. salmiana* leaves (ASL), (c) *A. tequilana* leaves (ATL), (d) *A. americana* heart (AAH), (e) poplar, and (f) switchgrass using different enzyme formulations at a total protein loading of 150 mg/g structural carbohydrates in raw biomass.** Details on enzymes formulations are given in Table S2. In the figures, 1500 represents Accellerase1500 cellulase, XY represents Accellerase XY xylanase, XC represents Accellerase XC xylanase, and P represents Multifect pectinase. A figure lists sugar release data from different agave samples, as well as poplar and switchgrass. Click here for file

Additional file 4 **Conditions applied for low severity hydrothermal pretreatments.** A table lists temperature and severity conditions of low severity hydrothermal pretreatments. Click here for file

Additional file 5 **X-Ray Diffraction (XRD) spectrum of Avicel PH 101 cellulose, 6-hour ball milled Avicel cellulose, *A. americana *** **leaves (AAL), and *A. americana *** **heart (AAH). A figure lists XRD data of agave samples, with comparison to Avicel.** Click here for file

Additional file 6 **Listing of plant cell wall glycan-directed monoclonal antibodies (mAbs) used for glycome profiling analyses (Figure **1**).** The groupings of antibodies are based on a hierarchical clustering of ELISA data generated from a screen of all mAbs against a panel of plant polysaccharide preparations^1,2^ to identify mAbs according to the predominant polysaccharides that they recognize. The majority of listings link to the Wall*Mab*DB plant cell wall monoclonal antibody database (http://www.wallmabdb.net) that provides detailed descriptions of each mAb, including immunogen, antibody isotype, epitope structure (to the extent known), supplier information. A figure lists antibodies used for glycome profiling experiments. Click here for file
