# Supplementary material for: Agave proves to be a low recalcitrant lignocellulosic feedstock for biofuels production on semi-arid lands
Source: Biotechnol Biofuels. 2014 Apr 4;7:50. doi: 10.1186/1754-6834-7-50 (PMC4022320; doi:10.1186/1754-6834-7-50)
Supplement: Additional file 1 — Chemical composition of agave, poplar, and switchgrass. A table lists chemical composition data of different agave samples, as well as poplar and switchgrass. [file 1754-6834-7-50-S1.docx]

Chemical composition of agave, poplar, and switchgrass.

| Chemical composition of agave, poplar, and switchgrass^1^ (g/100 g biomass) | | | | | | |
| --- | --- | --- | --- | --- | --- | --- |
|  | WSC^2^ | Glucose | Xylose | Galactose | Arabinose | K-lignin |
| AAL | 6.5 | 33.8 | 8.2 | 5.2 | 3.2 | 8.2 |
| ASL | 7.9 | 32.1 | 8.5 | 4.0 | 3.1 | 9.8 |
| ATL | 4.4 | 33.7 | 8.8 | 2.0 | 2.0 | 11.9 |
| AAH | 17.0 | 22.8 | 7.7 | 9.9 | 3.9 | 7.3 |
| Poplar^3^ | - | 51.4 | 22.9 | - | - | 23.4 |
| Switchgrass^3^ | - | 36.0 | 24.0 | - | - | 18.8 |
| ^1^Data reported are the mean values of three replicates based on oven dried material.  ^2^Water soluble carbohydrates  ^3^WSC, galactan and arabinan contents for poplar and switchgrass were not determined due to the low amount | | | | | | |
