# Supplementary material for: Agave proves to be a low recalcitrant lignocellulosic feedstock for biofuels production on semi-arid lands
Source: Biotechnol Biofuels. 2014 Apr 4;7:50. doi: 10.1186/1754-6834-7-50 (PMC4022320; doi:10.1186/1754-6834-7-50)
Supplement: Additional file 2 — Enzymes, formulations, and protein proportions of fungal enzyme cocktails applied for biomass hydrolysis. A table lists composition of enzyme cocktails which were used in the experiment for enzymatic hydrolysis of biomass samples. [file 1754-6834-7-50-S2.docx]

| Enzymes, formulations, and protein proportions of fungal enzyme cocktails applied for biomass hydrolysis | | | | |
| --- | --- | --- | --- | --- |
|  | Protein mass, % | | | |
| Enzyme | Accellerase^®^ 1500 | Accellerase^®^ XY | Accellerase^®^ XC | Multifect^®^ Pectinase |
| 1500 | 100 | - | - | - |
| 1500+XY | 75 | 25 | - | - |
| 1500+XC | 75 | - | 25 | - |
| 1500+P | 75 | - | - | 25 |
| 1500+XY+P | 75 | 12.5 | - | 12.5 |
| 1500+XC+P | 75 | - | 12.5 | 12.5 |
| 1500+XY+XC | 75 | 12.5 | 12.5 | - |
| Accellerase®1500: commercial mixture, mostly cellulase  Accellerase® XY: accessory enzyme, mostly xylanase  Accellerase® XC: accessory enzyme, xylanase with multiple hemicellulase activities  Multifect® pectinase: pectinase and multiple hemicellulase activities | | | | |
