# Supplementary material for: Agave proves to be a low recalcitrant lignocellulosic feedstock for biofuels production on semi-arid lands
Source: Biotechnol Biofuels. 2014 Apr 4;7:50. doi: 10.1186/1754-6834-7-50 (PMC4022320; doi:10.1186/1754-6834-7-50)
Supplement: Additional file 3 — Total sugar release from hydrothermal pretreatment (180C- 11.1 min) followed by enzymatic hydrolysis of (a) A. americana leaves (AAL), (b) A. salmiana leaves (ASL), (c) A. tequilana leaves (ATL), (d) A. americana heart (AAH), (e) poplar, and (f) switchgrass using different enzyme formulations at a total protein loading of 150 mg/g structural carbohydrates in raw biomass. Details on enzymes formulations are given in Table S2. In the figures, 1500 represents Accellerase1500 cellulase, XY represents Accellerase XY xylanase, XC represents Accellerase XC xylanase, and P represents Multifect pectinase. A figure lists sugar release data from different agave samples, as well as poplar and switchgrass. [file 1754-6834-7-50-S3.docx]

Total sugar release from hydrothermal pretreatment (180C- 11.1 min) followed by enzymatic hydrolysis of (a) A. *americana* leaves (AAL), (b) A. *salmiana* leaves (ASL), (c) A. *tequilana* leaves (ATL), (d) A. *americana* heart (AAH), (e) poplar, and (f) switchgrass using different enzyme formulations at a total protein loading of 150 mg/g structural carbohydrates in raw biomass. Details on enzymes formulations are given in Table S2. In the figures, 1500 represents Accellerase^®^1500 cellulase, XY represents Accellerase^®^ XY xylanase, XC represents Accellerase^®^ XC xylanase, and P represents Multifect^®^ pectinase.
