# Supplementary material for: Agave proves to be a low recalcitrant lignocellulosic feedstock for biofuels production on semi-arid lands
Source: Biotechnol Biofuels. 2014 Apr 4;7:50. doi: 10.1186/1754-6834-7-50 (PMC4022320; doi:10.1186/1754-6834-7-50)
Supplement: Additional file 4 — Conditions applied for low severity hydrothermal pretreatments. A table lists temperature and severity conditions of low severity hydrothermal pretreatments. [file 1754-6834-7-50-S4.docx]

| Conditions applied for low severity hydrothermal pretreatments | | |
| --- | --- | --- |
| Temperature (℃) | Reaction times for corresponding severities^1^ (min) | |
|  | Log Ro=3.0 | Log Ro=3.4 |
| 105 | 712.8 | - |
| 120 | 257.4 | - |
| 140 | 66.5 | 166.8 |
| 160 | 17.1 | 43.0 |
| 180 | 4.4 | 11.1 |
| ^1^Pretreatment severity is defined as $R_{0}=t\cdot e^{\frac{T-100}{14.75}}$, where t is in minutes and T in ^o^C | | |
