# Supplementary material for: Agave proves to be a low recalcitrant lignocellulosic feedstock for biofuels production on semi-arid lands
Source: Biotechnol Biofuels. 2014 Apr 4;7:50. doi: 10.1186/1754-6834-7-50 (PMC4022320; doi:10.1186/1754-6834-7-50)

X-Ray Diffraction (XRD) spectrum of Avicel PH 101 cellulose, 6-hour ball milled Avicel cellulose, A. *americana* leaves (AAL), and A. *americana* heart (AAH).


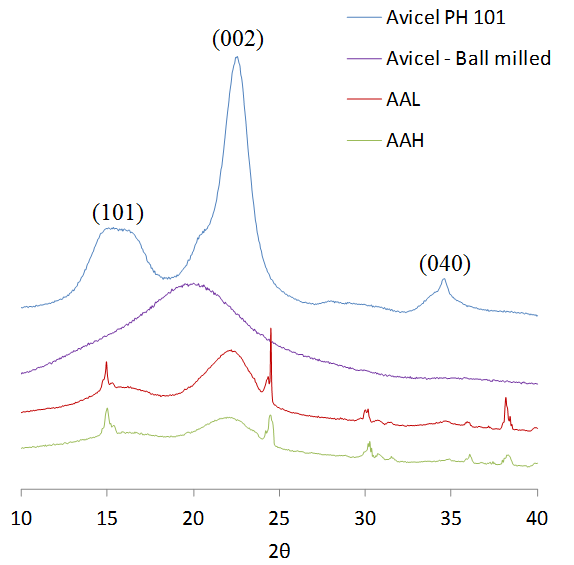

Supplement: Additional file 5 — X-Ray Diffraction (XRD) spectrum of Avicel PH 101 cellulose, 6-hour ball milled Avicel cellulose, A. americana leaves (AAL), and A. americana heart (AAH). A figure lists XRD data of agave samples, with comparison to Avicel. [file 1754-6834-7-50-S5.docx]
